# Motor imagery modulation of body sway is task-dependent and relies on imagery ability

**DOI:** 10.3389/fnhum.2014.00290

**Published:** 2014-05-08

**Authors:** Thiago Lemos, Nélio S. Souza, Carlos H. R. Horsczaruk, Anaelli A. Nogueira-Campos, Laura A. S. de Oliveira, Claudia D. Vargas, Erika C. Rodrigues

**Affiliations:** ^1^Instituto de Biofísica Carlos Chagas Filho, Universidade Federal do Rio de JaneiroRio de Janeiro, Brasil; ^2^Programa de Pós-Graduação em Ciências da Reabilitação, Centro Universitário Augusto MottaRio de Janeiro, Brasil; ^3^Departamento de Fisiologia, Universidade Federal de Juiz de ForaMinas Gerais, Brasil; ^4^Instituto D'Or de Pesquisa e EnsinoRio de Janeiro, Brasil

**Keywords:** center of gravity, postural control, motor imagery vividness, dynamic analyses, functional reaching

## Abstract

In this study we investigate to what extent the effects of motor imagery on postural sway are constrained by movement features and the subject's imagery ability. Twenty-three subjects were asked to imagine three movements using the kinesthetic modality: rising on tiptoes, whole-body forward reaching, and whole-body lateral reaching. After each task, subjects reported the level of imagery vividness and were subsequently grouped into a HIGH group (scores ≥3, “moderately intense” imagery) or a LOW group (scores ≤2, “mildly intense” imagery). An eyes closed trial was used as a control task. Center of gravity (COG) coordinates were collected, along with surface EMG of the deltoid (medial and anterior portion) and lateral gastrocnemius muscles. COG variability was quantified as the amount of fluctuations in position and velocity in the forward-backward and lateral directions. Changes in COG variability during motor imagery were observed only for the HIGH group. COG variability in the forward-backward direction was increased during the rising on tiptoes imagery, compared with the control task (*p* = 0.01) and the lateral reaching imagery (*p* = 0.02). Conversely, COG variability in the lateral direction was higher in rising on tiptoes and lateral reaching imagery than during the control task (*p* < 0.01); in addition, COG variability was higher during the lateral reaching imagery than in the forward reaching imagery (*p* = 0.02). EMG analysis revealed no effects of group (*p* > 0.08) or task (*p* > 0.46) for any of the tested muscles. In summary, motor imagery influences body sway dynamics in a task-dependent manner, and relies on the subject' imagery ability.

## Introduction

Human postural control refers to the ability to sustain an upright standing position by counteracting gravity-toppling torque and stabilizing the overall configuration of a multi-segmented body. Postural control relies on the integration of multiple sensory systems and on the active generation of muscle force along the body axis (Horak and Nashner, 1986; Peterka, 2002). Recent investigations have revealed that postural control is also influenced by high-level cognitive processes such as the mental simulation of a movement or motor imagery (Rodrigues et al., 2003, 2010; Grangeon et al., 2011). Motor imagery can be defined as a particular mental state in which dynamic, time-evolving images of actions are generated without a corresponding overt execution (Jeannerod, 1994).

It has been consistently demonstrated that postural sway is markedly changed when standing subjects imagine either whole body (Rodrigues et al., 2010; Grangeon et al., 2011) or a finger-to-thumb opposition task (Grangeon et al., 2011). The effects of motor imagery on postural sway are more pronounced when kinesthetic cues (i.e., sensory information about body position and motion) are employed, in contrast to the visual, third-person imagery of the same movement (Rodrigues et al., 2003, 2010; Grangeon et al., 2011). This increase in postural sway during kinesthetic motor imagery could be explained in terms of changes in body state estimates (its position and velocity) through forward internal models (Davidson and Wolpert, 2005; Jeannerod, 2006), and/or imagery-selective modulation of muscle spindle sensitivity (Gandevia et al., 1997). Additionally, the modulation of the motor system's activity during motor imagery could also account for the changes in postural control.

Indeed, motor imagery modulates several motor neural networks, including cortical (e.g., Fadiga et al., 1999; Hétu et al., 2013), and spinal circuitries (Bonnet et al., 1997; Li et al., 2004; Aoyama and Kaneko, 2011). In addition, the effects of motor imagery on the motor system show a high degree of task-specificity: modulation of autonomic responses during imagery is proportional to the effort of the imagined movement (Decety et al., 1991); increases in corticospinal excitability occur specifically in muscles involved in the imagined action (Fadiga et al., 1999; Fourkas et al., 2006; Stinear et al., 2006); and muscle responses to stretch reflexes are higher during stronger plantar flexion imagery than during weaker plantar flexion imagery (Bonnet et al., 1997). In addition, the biomechanical constrains of an action (Jeannerod, 2006), as well as its temporal curse (Decety et al., 1989), are also reflected in the mental image of a movement.

Given that motor imagery activates the motor system in a task-specific manner, and embodies some of the tasks constrains, it is possible that the corresponding changes in body sway also hold for task postural requirements. The pattern of postural adjustments preceding or following movement is usually dependent of the action characteristics. For example, during the execution of a rise on tiptoe movement, a significant forward-backward motion of the body center of gravity can be observed (Nardone and Schieppati, 1988); likewise, when a subject is instructed to “reach sideward as far as possible,” large lateral trunk motion is required (Verheyden et al., 2011) and increases in lateral center of pressure (COP) displacement occurs (Brauer et al., 1999). Although there is some suggestive evidence that motor imagery modulates postural sway in a task-dependent manner (Rodrigues et al., 2003, 2010; Grangeon et al., 2011; Boulton and Mitra, 2013) this issue remains largely unexplored.

In the present study, we investigate whether postural features of an imagined action influence postural responses to motor imagery. Because the effects of motor imagery on the motor system greatly depend on the individual's capability to imagine an action (Guillot et al., 2008; Olivetti Belardinelli et al., 2009), we also ask whether imagery modulation of postural sway rely on the subject' imagery ability. Imagery of three movements was employed, each differing in terms of magnitude and direction of postural requirements: rising on tiptoes, whole-body forward reaching, and whole-body lateral reaching. Imagery ability was assessed through motor imagery vividness scores that were reported after each imagery trial. Change in the center of gravity (COG) position and velocity (i.e., COG dynamics; Pai and Patton, 1997; Pai et al., 2000) was assessed during motor imagery task. Our hypothesis was that: (i) the effect of motor imagery on body sway is the result of the embodiment of the postural features of the action on the motor image content; and (ii) the occurrence of imagery modulation of body sway will be evident for those subjects with higher imagery ability. Accordingly, we expect that highly skilled imaginers' show specific changes in COG dynamics in the forward-backward or lateral directions depending on the imagined movement. Once motor imagery is largely applied in clinical settings for, e.g., rehabilitation after stroke (Langhorne et al., 2009), and has been already used for balance training purposes in young persons (Choi et al., 2010), and in elderly population (Fansler et al., 1985; Hamel and Lajoie, 2005), understanding how the mental simulation of an action affects COG dynamics could improve the use of motor imagery approach for balance rehabilitation.

## Methods

### Participants

Twenty-three participants were tested (11 males; age range 20–38 years; 153–189 cm height; and 53–74 kg weight). All subjects were right-handed, as assessed by the Edinburgh Handedness Inventory. There were no reports of neurological or musculoskeletal disease that could impair maintenance of their standing posture. Written informed consent was provided prior to participation in the study, and the experiment was approved by the local ethical committee (process 479.056) and conformed to the latest amendments set by the Declaration of Helsinki.

### Experimental procedures

**Figure 1:**
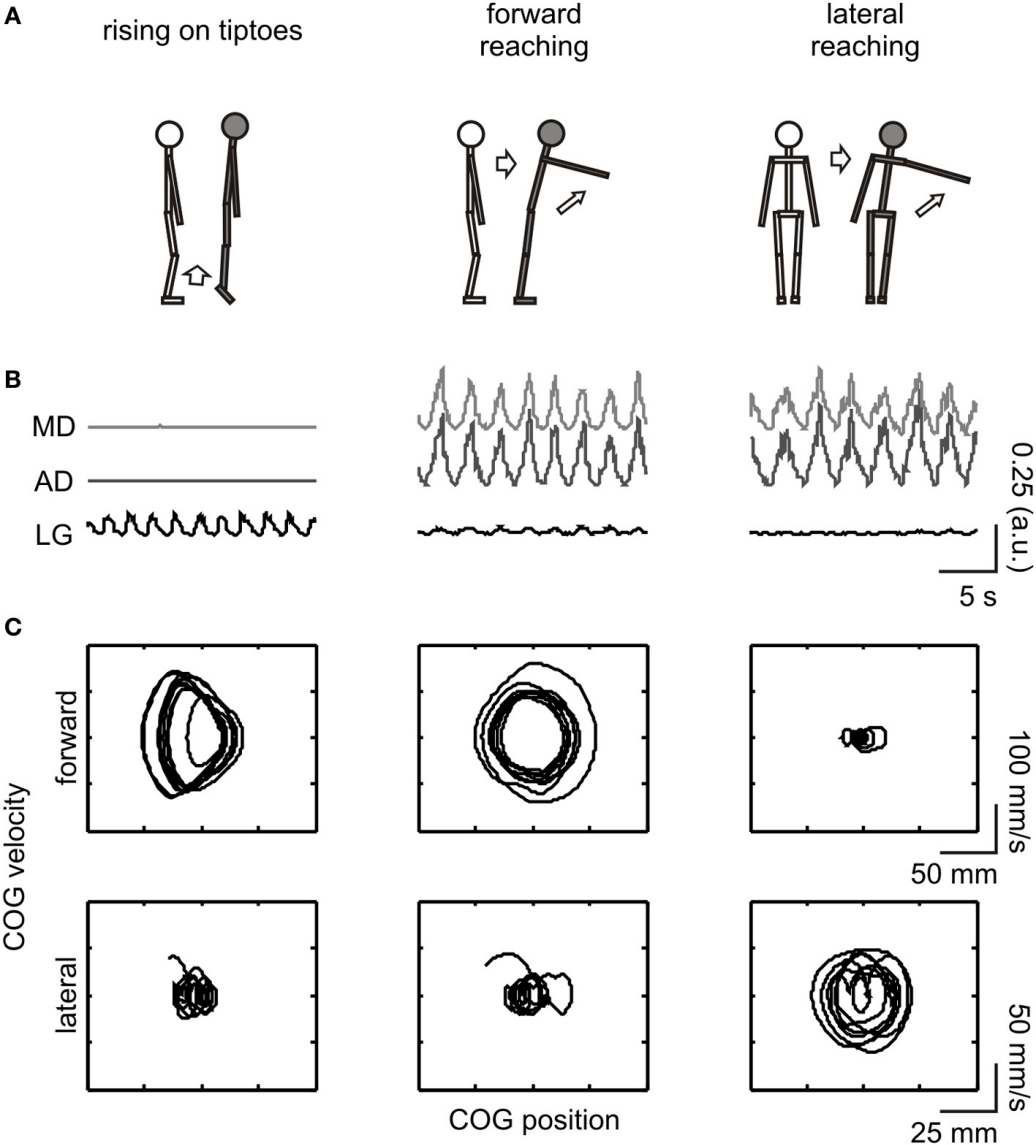
**Schematic illustration of the experimental procedure**. From top to bottom: **(A)** depiction of the movements executed and imagined; **(B)** full-wave rectified EMG from the evaluated muscles [MD (light gray line) and AD (dark gray line) corresponds to the middle and anterior portion of the deltoid muscle, respectively, while LG (black line) refers to the lateral gastrocnemius]; **(C)** COG phase-plane plot in the forward-backward (upper row) and lateral (lower row) directions. EMG and COG data are from an execution trial of a representative subject (#03). For illustrative purposes, data are shown for a short (20 s) epoch. COG dynamics changed in the forward-backward direction during execution of rising on tiptoes and forward reaching tasks and in the lateral direction during the lateral reaching task. The LG and deltoid muscles were highly activated during the rising on tiptoes and both reaching tasks, respectively.

Subjects were positioned over a force platform, with feet closely together and arms relaxed alongside their body. A brief period of adaptation was given for each participant before the experimental session. Subjects were first instructed to stand quietly for 45 s with the eyes closed (control task). Next, the subjects were asked to execute and imagine the following tasks: reaching forward with their right hand; reaching laterally with their right hand; and rising on tiptoes (Figure 1A). The execution and imagery trials were performed in two different blocks. The order of the tasks was randomly assigned for each subject, and the same order was applied for execution and imagery blocks. Execution trials were always conducted first to characterize the COG dynamics of the tested movements. In this case, subjects were instructed to keep their eyes open and to (i) rise on their tiptoes or (ii) to reach (forward or laterally) as far as possible, without bending or twisting the trunk or changing the base of support. For imagery trials, the eyes were kept closed, and subjects were asked to imagine the required movement in the kinesthetic modality,—i.e., they should “feel themselves” performing the movement. Subjects were free to perform or imagine the required movement at their preferred speed. A unique 45 s trial was used for each task, with 1–2 min inter-task intervals. Subjects' feet borders were marked on the platform to ensure that the same position was adopted among trials. Execution and imagery trials initiated and ceased upon the issuing of an auditory cue.

After each trial, subjects were asked about the number of movements they performed. These values were compared to test for temporal equivalences between execution and imagery trials. After the imagery trials, subjects were asked about the intensity of the sensation evoked by the imagined movement, using a 5-point scale designed by Malouin et al. (2007). In this scale, 1 corresponds to “no sensation” and 5 corresponds to “as intense as executing the action.” The imagery vividness score was taken as a measure of the subject's imagery ability, with higher scores corresponding to an increased ability to mentally imagine an action. To test the influence of imagery ability on COG variability, subjects were categorized into two groups: those whose scores were equal to or less than 2 (“mildly intense” imagery) in at least one task were grouped in the low score category (LOW group), and those subjects whose scores were equal to or higher than 3 (“moderately intense” imagery) for all tasks were grouped in the high score category (HIGH group).

### Data acquisition

Feet center of pressure (COP) coordinates were calculated from the ground reaction forces measured with a force platform (AccuSway^PLUS^, AMTI). Force signals were sampled at 50 Hz with a 5 Hz cutoff anti-aliasing filter.

Electromyography (EMG) of the shoulder and ankle muscles involved in the tested movements (Figure 1B) was assessed to confirm that subjects were not executing the required action during imagery trials. The EMG was measured through bipolar surface electrodes (Ag/AgCl, 8 mm diameter; 20 mm inter-electrode distance). After skin preparation by abrasion and shaving, the electrodes were placed on the middle (MD) and anterior (AD) portions of the deltoid muscle, and in the lateral gastrocnemius (LG), using the recommendations of Barbero et al. (2012). Briefly, the electrodes were placed at half of the distance between the coracoids process and the deltoid tuberosity (MD); at the proximal 1/5 of the distance between the acromion and the lateral epicondyle (AD); and at the proximal 1/4 of the distance between the popliteal fossa and the lateral border of the Achilles tendon (LG). The EMG signal was amplified 2000 times and digitized at 2000 Hz (10–500 Hz bandpass filtered, MP150WS, BIOPAC System).

### Data processing

Before data processing, the first 5 s of each trial were discarded and the remaining 40 s was used for analyses. The dynamics of body sways was accessed through COG variability, measured as proposed by Riley et al. (1995). COG coordinates were obtained through low-pass filtering the COP time series (Caron et al., 1997), and the COG velocity was computed by differentiating the COG displacement. Riley's directional stability (DS) value is a unitless parameter that provided an estimate of the amount of variability in COG dynamics in a particular direction (forward-backward or lateral), considering the fluctuations in COG position and velocity. The DS value was calculated as the square root of the summed variances of the COG position and velocity measured for the whole 40 s trial, separately for the forward-backward (DS_FWB_) and lateral direction (DS_LAT_). Fluctuations in muscle activity were estimated through the full-wave rectified, low-pass filtered EMG envelope (5 Hz cutoff, 2nd order Butterworth filter); then, the coefficient of variation (COV) was calculated for the entire 40 s trial. Given that COV values are inherently normalized with respect to the mean value, these values were unlikely to be affected by spurious changes in EMG amplitude typically resulting from anatomical differences between subjects, e.g., fat thickness (Farina et al., 2002).

### Statistical analyses

A repeated measure ANOVAs was applied to check for differences among execution tasks (forward reaching, lateral reaching, and rising on tiptoes) on COG directional stability values in both direction, as well as on the EMG modulation of the deltoids and lateral gastrocnemius muscles. Subjective measures were analyzed separately for HIGH and LOW group. The differences in the number of movements executed or imagined among tasks were tested with the paired *t*-test, and the kinesthetic vividness scores given after each imagery trial were compared through One-Way ANOVA. A repeated measure Two-Way ANOVA was applied to test for a main effect of group (HIGH vs. LOW) and task (control and imagery trials of rising on tiptoes, forward reaching, and lateral reaching tasks). The same procedure was employed for testing COV values. When a significant F was attained, Tukey-HSD *post-hoc* analysis was applied for paired comparisons. The threshold for statistical significance was set at *p* = 0.05. Data are presented as the mean ± standard error of mean (s.e.m.).

## Results

### Motor execution measures

Significant differences in forward-backward [*F*_(2, 44)_ = 86.8, *p* < 0.001] and lateral [*F*_(2, 44)_ = 11.7, *p* < 0.001] COG variability were observed among tasks (see Figure 1C for COG dynamics of a representative subject). A progressive increase in forward-backward COG variability was observed from lateral reaching (DS_FWB_ = 11.7 ± 0.7) to forward reaching (41.3 ± 4.5), with rising on tiptoes showing the greatest variability (72.6 ± 3.6; all posttest *p* < 0.001). Accordingly, lateral reaching execution promoted larger lateral COG variability (DS_LAT_ = 22.2 ± 1.7) than rising on tiptoes (13.7 ± 1.6; *p* < 0.001) and forward reaching (13.2 ± 0.5; *p* < 0.001). No difference between rising on tiptoes and forward reaching was observed (*p* = 0.82). Figure 1B highlights the specific muscle EMG pattern for the three tasks during execution. As can be observed, the LG muscle was highly activated during the rising on tiptoes, and deltoid muscles show increased EMG fluctuations when both reaching tasks were performed; statistical analyses confirm the main effect of tasks for the three muscles (ANOVAs *p* < 0.01). Average COV values of both deltoid muscles range 87–110% during forward and lateral reaching execution, while remains around 20% during rise on tiptoes execution (posttest *p* < 0.0002). On the other hand, lateral gastrocnemius activity was highly modulated during rise on tiptoes execution (COV = 98 ± 3%) than during forward reaching (63 ± 5%) or lateral reaching execution (42 ± 4%; posttest *p* < 0.0004).

### Subjective reports and group characteristics

Based on imagery vividness score distribution, 10 subjects were categorized in the LOW group (scores = 2, corresponding to “mildly intense” sensation, for at least one task), and the remaining 13 were categorized in the HIGH group (scores = 3, correspondent to a “moderately intense” sensation, for all tasks). There was no difference between groups concerning gender distribution (5/10 subjects were male in the LOW group, and 6/13 subjects were male in the HIGH group; no difference in male/female distribution; χ^2^ = 0.74, *p* = 0.39). The same result was obtained for age (mean ± SD; 24 ± 3 years for LOW and 25 ± 5 years for HIGH vividness group; *t* = −0.6, *p* = 0.53), height (173 ± 8 cm for LOW and 168 ± 8 cm for HIGH vividness group; *t* = 1.3, *p* = 0.21), body mass (65.9 ± 10.9 kg for LOW and 60.1 ± 8.2 kg for HIGH vividness group; *t* = 1.5, *p* = 0.16), and body mass index (22.1 ± 3.8 kg/m^2^ for LOW and 21.2 ± 2.3 kg/m^2^ for HIGH vividness group; *t* = 0.7, *p* = 0.51). Subsequent analyses were performed on each group to check for the potential influence of motor imagery ability on postural responses among tasks.

No difference in the number of executed and imagined movements was observed between groups (*p* > 0.05; Supplemental Table 1), suggesting similar motor imagery temporal characteristics. In addition, no differences among tasks were found for the vividness scores, independent of the analyzed group [HIGH group *F*_(2, 24)_ = 0.09, *p* = 0.9; LOW group *F*_(2, 18)_ = 2.9, *p* = 0.08; Supplemental Table 2].

### COG dynamics during motor imagery

**Figure 2:**
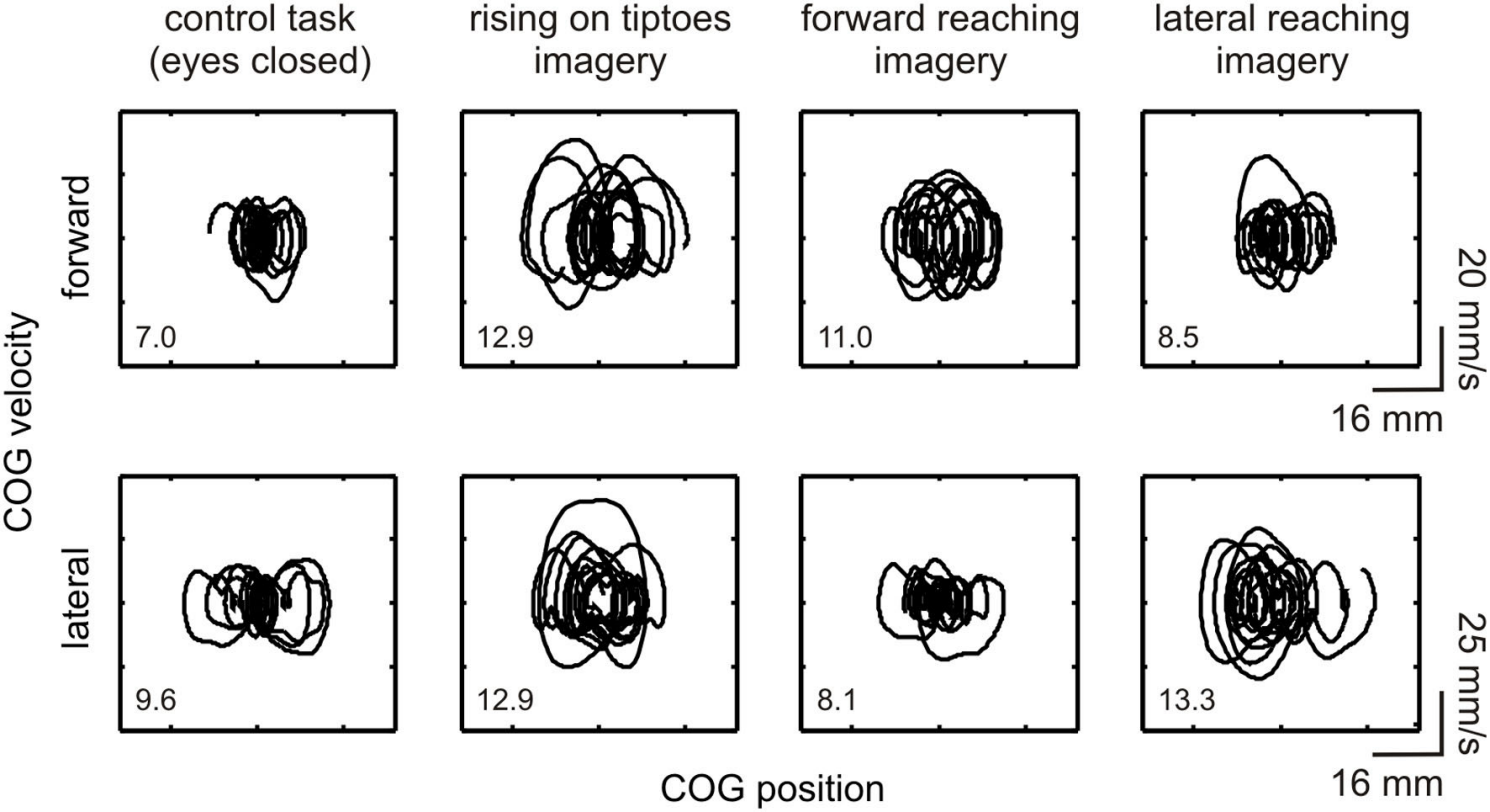
**Effect of motor imagery on COG variability**. COG phase-plane plots from a representative subject (#11) are shown during control and imagery tasks (as indicated under each column). Plots are shown for the forward-backward (indicated as “forward,” upper plots) and lateral (lower plots) directions. Correspondent directional stability values (DS_FWB_ and DS_LAT_) are presented as an inset. Large COG variability was observed in the forward-backward direction during the rising on tiptoes and forward reaching imagery tasks; conversely, changes in COG variability in the lateral direction occurred in the rising on tiptoes and lateral reaching imagery tasks.

The general effect of motor imagery on COG dynamics is presented in Figure 2 as a phase-plane plot from one representative subject (#11). Increases in forward-backward COG variability were noted for the rising on tiptoes and forward reaching imagery tasks compared with the control task and lateral reaching imagery (see DS values in the figure inset). As expected, a different behavior emerges in the lateral direction, with large COG variability being attained during lateral reaching imagery and rising on tiptoes tasks, and similar variability in the control and forward reaching imagery tasks. These results point to specific changes in body sway dynamics depending on the postural requirements of the imagined movement.

**Figure 3:**
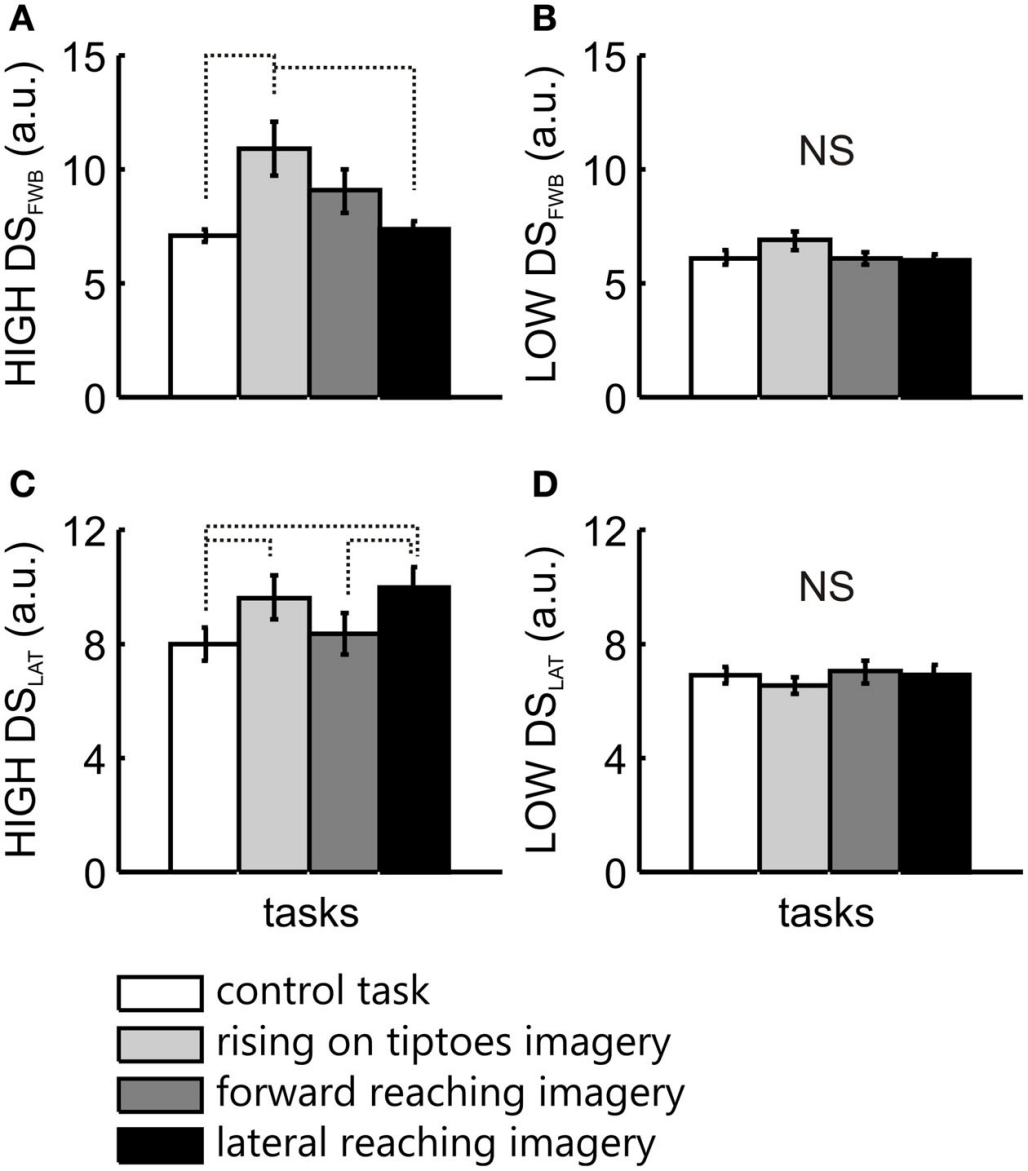
**COG variability analysis for the two imagery vividness groups**. The mean ± s.e.m. of directional stability in the forward-backward (DS_FWB_) and lateral (DS_LAT_) directions are presented for the HIGH **(A,C)** and the LOW **(B,D)** imagery vividness groups, respectively. Data are shown for control task (white bars), rising on tiptoes imagery (light gray bars), forward reaching imagery (dark gray bars), and lateral reaching imagery (black bars). Significant differences are indicated by dotted lines.

For comparisons between groups, a Two-Way ANOVA was applied, with LOW and HIGH vividness groups as the between-factor, and tasks as the within-factor. Statistical analysis in the forward-backward direction revealed a main effect of task [*F*_(3, 63)_ = 4.6, *p* = 0.006], with no significant interaction [*F*_(3, 63)_ = 2.1, *p* = 0.1]. Posttest analysis of task effects showed a significant difference between control task and rise on tiptoes imagery (*p* = 0.004), and between rise on the tiptoes and lateral reaching imagery (*p* = 0.006). There was also a significant group effect [*F*_(1, 21)_ = 6.28, *p* = 0.02]. To elucidate the specific group effect of imagery ability on forward-backward COG variability, separate ANOVAs were applied for HIGH and LOW vividness group, with task as the within-factor. There was a main effect of task in the HIGH group [*F*_(3, 36)_ = 4.8, *p* = 0.007]. The rising on tiptoes imagery task was different from the control (*p* = 0.01) and the lateral reaching imagery (*p* = 0.02), with no other differences among tasks (*p* > 0.33; Figure 3A). Conversely, the LOW group showed comparable DS_FWB_ values across all tasks [*F*_(3, 27)_ = 1.0, *p* = 0.41; Figure 3B].

In the lateral direction a main effect for task [*F*_(3, 63)_ = 3.3, *p* = 0.03] and a significant interaction between group and task [*F*_(3, 63)_ = 5.1, *p* = 0.003] was observed, with no main effect for group [*F*_(1, 21)_ = 3.8, *p* = 0.06]. Posttest analyses revealed changes in COG variability only in the HIGH group. DS_LAT_ obtained in the control task was different from that observed in the rising on tiptoes imagery (*p* = 0.01) and the lateral reaching imagery tasks (*p* = 0.001; Figure 3C). In addition, forward reaching imagery was different from lateral reaching imagery (*p* = 0.02; Figure 3C); again, the LOW group showed no significant difference for any task (>0.98; Figure 3D).

### EMG measures

A repeated measure Two-Way ANOVA was applied for each muscle to check for differences in EMG activity between groups (LOW and HIGH) and tasks (control task, forward reaching, lateral reaching, and rising on tiptoes imagery). There was no main effect of group (ANOVAs *p* > 0.08), tasks (*p* > 0.46) and no significant interaction (*p* > 0.39) for any of the evaluated muscles (Table 1).

**Table 1:** **Modulation of muscle activity during control and imagery tasks**.

	**CO**	**RTI**	**FRI**	**LRI**
**HIGH SCORE GROUP (*n* = 13)**				
MD	8.2 ± 1.4	8.6 ± 1.8	8.1 ± 1.4	8.3 ± 1.8
AD	9.2 ± 1.6	10.8 ± 2.0	12.1 ± 2.8	9.9 ± 1.8
LG	24.0 ± 4.1	27.7 ± 5.7	23.8 ± 4.6	26.4 ± 4.4
**LOW SCORE GROUP (*n* = 10)**				
MD	11.6 ± 1.5	11.6 ± 2.0	12.7 ± 2.8	14.1 ± 2.6
AD	8.1 ± 1.0	8.0 ± 1.2	8.2 ± 1.3	8.6 ± 1.3
LG	24.9 ± 4.9	23.5 ± 4.6	23.0 ± 5.0	20.7 ± 2.9

EMG envelope COV (%) values was presented for each of the two groups (HIGH and LOW imagery vividness score) during control task (CO), rising on tiptoes (RTI), forward reaching (FRI), and lateral reaching (LRI) imagery tasks. MD, middle portion of deltoid; AD, anterior portion of deltoid; LG, lateral gastrocnemius. Data are presented as the mean ± s.e.m.

## Discussion

The aim of the present study was to evaluate whether motor imagery modulation of body sway relates to the postural requirements of the imagined task, and whether the subject's imagery ability influences postural responses to motor imagery. Our findings reveal that (i) during motor imagery, COG dynamics are modulated in a task-specific manner, showing increased variability in the forward-backward or lateral direction depending on the postural requirement of the imagined movement; and (ii) the modulation of COG dynamics relies on the subject's motor imagery ability. We now discuss these findings and some possible mechanisms that could account for them.

### Motor imagery modulates body sway in a task-dependent manner

Motor imagery promotes increases in COG variability depending on the postural requirements of the imagined action. The effects of imagery on postural control scaled with the direction of COG changes when subjects executed the rising on tiptoes and lateral reaching tasks. Rising on tiptoes imagery lead to higher forward-backward COG variability than the control task; similarly, rising on tiptoes and lateral reaching imagery increased lateral COG variability (see Figures 3A,C). The effect of rising on tiptoes imagery on COG dynamics resembled that observed previously for COP fluctuations (Rodrigues et al., 2003, 2010), where both forward-backward and lateral sways increased significantly.

These specific modulations in COG dynamics in the rising on tiptoes and lateral reaching imagery are indicative of some correspondence between postural adjustments during actual performance and those observed during motor imagery: rising on tiptoes execution is accompanied by a striking reduction in the base of support, large forward-backward displacement of the COG (Nardone and Schieppati, 1988) and greater COG variability (2–6-fold higher than both reaching execution tasks); accordingly, execution of lateral reaching movements requires a large lateral trunk motion (Verheyden et al., 2011) and COP displacement (Brauer et al., 1999), along with a large increase in lateral COG variability (~2-fold higher than rising on tiptoes and forward reaching task). Comparing this COG dynamics pattern with that evoked during motor imagery, we suggest the existence of some execution-imagery “postural correspondence.”

Nevertheless, considerable postural adjustments also precede and follow a forward reaching motion (Stapley et al., 1998; Cavanaugh et al., 1999). Indeed we observed increased forward-backward COG variability during the execution of this task (Figure 1C). Based on the execution-imagery “postural correspondence” assumption, the lack of an effect of forward reaching imagery on COG variability seems counterintuitive. There are some factors that could influence these results: one was related to the instruction to “reach as far as possible, without bending or twist the trunk” (i.e., constraining trunk motion), because restriction in trunk motion could affect the performance of the forward reaching task (as observed in the aging processes; see Cavanaugh et al., 1999). However, the large increases in COG variability observed during forward reaching execution suggest that the instruction was of little influence, at best. Another explanation comes from the fact that forward reaching task rely more on trunk motion than the lateral reaching task. Although small trunk motion occurs during quiet standing position (Creath et al., 2005) it could be compensated by changes in the motion of other body segments (Hsu et al., 2007). Based on that, one could suppose that trunk motion produced by motor imagery is barely reflected on COG dynamics, resulting only in a small, non-significant increase in DS_FWB_ values (see Figure 3A). On the other hand, the impact of trunk motion is expected to be smaller for the lateral reaching tasks because for narrower stances (i.e., the feet closely together) body sway in the lateral direction results predominantly from ankle motion rather than hip motion (Day et al., 1993; Winter et al., 1996). Therefore, it seems that mentally simulating a whole-body reaching movement in a narrow stance could restrain observable changes in forward-backward, but not lateral COG variability.

Altogether, the motor imagery task-specific modulations of body sway dynamics corroborate our hypothesis that the modulation of body sway during motor imagery is constrained by the postural features of the actual movement. This task-dependent effect of motor imagery on postural control is similar to that recently reported by Boulton and Mitra (2013). By using kinematic measures of head and trunk displacement, these authors found differences between forward and lateral trunk displacement depending on the imagined arm motion. However, their results actually demonstrated a reduction in head/trunk sway during motor imagery in relation to quiet standing, their baseline condition, in opposition to the increases in COG variability observed in the present study (see Figure 2). Differences in methodological setup, movements employed and motor imagery instructions could explain this apparently contradictory result. Otherwise, as several patterns of joint coordination along longitudinal body axis could be employed to stabilize body center of mass position (Hsu et al., 2007), the assessment of displacement of a single body segment gives distinct information about postural control changes, compared to the measurement of COP and COG sway.

In the present study, COG dynamics were measured during motor imagery in an upright stance position. As upright body stability depends on the active maintenance of COG inside the limits of the base of support, both horizontal position and velocity must be effectively controlled (Pai and Patton, 1997; Pai et al., 2000); otherwise, the subjects are at risk of falling. Quantification of postural dynamics, particularly its variability (the amount of fluctuation in COG or COP position and velocity), has been employed to demonstrate changes in postural control owing to altered stance (Riley et al., 1995) and surface compliance (Negahban et al., 2009), or due to pathological conditions such as vestibular disorders (Riley et al., 1995), anterior cruciate ligament injury (Negahban et al., 2009), low back pain (Salavati et al., 2009), and cerebellar degeneration (Hudson and Krebs, 2000). Our results provide further evidence that postural dynamics analysis can characterize changes in postural control related to motor imagery in healthy subjects.

### Body sway modulation relies on subject's motor imagery ability

In our group analysis (HIGH and LOW vividness groups), neither differences in temporal features (i.e., the number of movement executed or imagined) nor the vividness of a particular imagined movement could be regarded as confounding factors because no significant differences were obtained among the tasks (see section Subjective Reports and Group Characteristics). Comparable results were also obtained by Rodrigues et al. (2010), implying that the modulation of postural control may be related to motor imagery itself and, in the case of the present study, to the demanding postural aspects of the imagined action. However, the fact that changes in COG dynamics were observed in the HIGH group but not in the LOW group indicates that the effect of imagery on postural control relies on the subject's imagery ability.

The imagery ability of a subject has previously been related to the level of activation of the motor system in neuroimaging studies (Guillot et al., 2008; Olivetti Belardinelli et al., 2009; Lorey et al., 2011). Using several tools to classify motor imagery ability (such as the imagery vividness or the easiness to imagine scales), previous studies reported greater activation of motor related brain areas, such as the posterior parietal and premotor cortices, in subjects classified as skilled imaginers or reporting a high vividness score (Guillot et al., 2008; Olivetti Belardinelli et al., 2009). Indeed, there is a parametric association between imagery vividness and the degree of activation of the parieto-premotor circuitry (Lorey et al., 2011). This higher-level activation of the motor system by skilled imaginers could promote (i) larger changes in forward internal model estimates during imagery, or (ii) could be related to stronger subliminal activation of the motor system. While there was no evidence for the first, TMS studies corroborated the second by showing that corticospinal excitability is highly modulated in skilled imaginers, compared with poor, unskilled ones (Fourkas et al., 2008; Williams et al., 2012). Following this, our proposed execution-imagery “postural correspondence” seems to be influenced by imagery abilities: the changes in COG dynamics are more evident in highly skilled imaginers and less evident in imaginers with poorer skills.

### Possible mechanisms for the effect of motor imagery on postural control

EMG activity of the calf and shoulder muscles was measured to confirm that subjects were not executing the required action (rising on tiptoes or reaching movement) during imagery trials. As expected, no modulation of EMG activity was observed for any of the evaluated muscles during the imagery trials (see Table 1). The lack of modulation of EMG amplitude measures during motor imagery found in the present and in a previous study (Rodrigues et al., 2010) was consistent with the idea that subjects are not performing actual movements during imagery trials.

Besides the lack of EMG amplitude changes during motor imagery in upright standing subjects, the overt effect of imagery on postural dynamics must be associated with the modulation of motor system activity. Indeed, motor imagery has a significant effect on several neural networks, including cortical (e.g., Fadiga et al., 1999; Hétu et al., 2013), spinal (Bonnet et al., 1997; Li et al., 2004; Aoyama and Kaneko, 2011) and autonomic circuitries (Decety et al., 1991, 1993; Demougeot et al., 2009). Notably, motor imagery modulation of the motor system is related to the features of the imagined action, such as the level of overall effort (e.g., Decety et al., 1991, 1993; Bonnet et al., 1997) and the muscular activation pattern (Fadiga et al., 1999; Fourkas et al., 2006). Our results on postural sway follow these previous observations, as COG variability specifically changes in one direction or another depending on the postural features of the imagined task.

Few investigations have dealt with the effects of motor imagery on overt motor behavior. In one of these studies, Wexler et al. (1998) found that subjects exhibit faster times and fewer errors in a manual task when they simultaneously imagined a compatible action compared with a incongruent imagery task; similar results were obtained by Ramsey et al. (2010), who showed that motor imagery of an incongruent action slows down the subsequent performance of a reach-and-grasp action. Conversely, incongruent posture of the imaginer's hand had a significant impact on motor imagery, either by slowing imagery performance (Sirigu and Duhamel, 2001; Vargas et al., 2004) or by reducing imagery modulation on corticospinal excitability (Vargas et al., 2004). These studies pointed to some degree of integration between body states and mental imagery, possibly related to the overlapping activation of neural circuitries and/or cognitive states engaged both in action execution and motor imagery. The overlap between motor cognitive states and motor execution has been described both in conceptual (Prinz, 1997; Schütz-Bosbach and Prinz, 2007) as well as in neural terms (Jeannerod, 2001). Our results of a task-dependent modulation in COG dynamics could come from this type of integration, and the proposed execution-imagery “postural correspondence” could emerge from an execution-imagery “neural/cognitive correspondence.”

How this “neural/cognitive correspondence” affects the postural behavior of upright standing subjects? The effect of motor imagery on postural control has been discussed in terms of changes in body state estimates through forward internal models evoked during imagery (see Discussion in Rodrigues et al., 2010; Grangeon et al., 2011). Indeed, Rodrigues and coworkers (2010) have proposed that forward models generated during motor imagery could potentially change muscle spindle sensitivity by fusimotor neuron activation, then modulating postural sway. Here we present an alternative proposition for the overt effect of motor imagery on body sway, also based on its direct effect on the motor system's activity. It is possible to speculate that motor imagery leads to a partial or weak activation of motor system, inducing a subliminal modulation of spinal motoneurons, bringing its membrane potential close to discharge threshold; this subliminal “imagery fringe” explains why there was no change in muscle EMG amplitude while increased responses were obtained after transcranial magnetic (Fadiga et al., 1999; Stinear et al., 2006) and peripheral nerve stimulation (Gandevia et al., 1997), as well as tendon stretching (Bonnet et al., 1997). The exact mechanism of this partial activation (or partial inhibition) is still debatable (Jeannerod, 1994; Guillot et al., 2012). Whatever the neural mechanisms related with it, one could suggest that this subliminal modulation could turn into a supraliminal activation when the motor system is already active, as during upright standing. As a consequence, this supraliminal modulation promoted by concurrent motor imagery and postural motor activation could lead to overt changes in postural sway.

While plausible, this change in motor system activity is not translated as increased EMG activity (see Table 1). One possible explanation is that, although related to the net motor unit properties (i.e., its recruitment and discharge rate), surface EMG amplitude usually underestimates the activity of motor neuron pools because of the amplitude cancellation effect (reviewed in Farina et al., 2004). Thus, modulation of muscle activity below the detection threshold of the employed methodology cannot be excluded.

### Practical implications

In the recent years, motor imagery has been extensively applied for rehabilitation purposes (Schuster et al., 2011; Malouin et al., 2013), and several benefits of its use have been reported (Langhorne et al., 2009). Motor imagery training, or mental practice, has already been employed to improve balance skills in elderly (Fansler et al., 1985; Hamel and Lajoie, 2005) and in young healthy subjects (Choi et al., 2010), and showed the potential of mental practice as a balance disorder treatment. The present study provides evidence that the effect of motor imagery on postural sway is tightly associated with the postural features of the imagined action. Based on our results, a few suggestions can be made about motor imagery use in clinical and rehabilitation settings: (i) in line with the task-dependent influence of motor imagery on postural control, we recommend that mental practice should be accomplished with imagined movements that challenge specific balance issues (e.g., whole-body lateral reaching); (ii) given that moderate to high imagery vividness is necessary for the overt postural effects of motor imagery, the imagery ability of patients must be checked before enrollment in mental practice training.

## Conclusions

In conclusion, during the mental rehearsal of an action, all aspects of the imagined movement are potentially embodied, including the accompanying postural adjustments. This embodiment of movement's postural features during motor imagery results in modulations on body sway that resemble those required for actual execution of the action. In addition, overt postural changes during motor imagery depend on the vividness of the generated kinesthetic images; the better the assessment of perceptual-motor images, the more prominent influence of imagery on postural control.

### Conflict of interest statement

The authors declare that the research was conducted in the absence of any commercial or financial relationships that could be construed as a potential conflict of interest.
