# Supplementary material for: Motor imagery modulation of body sway is task-dependent and relies on imagery ability
Source: Front Hum Neurosci. 2014 May 8;8:290. doi: 10.3389/fnhum.2014.00290 (PMC4021121; doi:10.3389/fnhum.2014.00290)
Supplement: Supplementary file 1 [file DataSheet1.DOCX]

**Supplementary Table 1**

| **SUPPL. TABLE 1 \| Temporal equivalence estimated from the number of executed and imagined movements performed** | | | |
| --- | --- | --- | --- |
| **HIGH score group (n=13)** | | | |
|  | **execution** | **imagery** | **p-value** |
| **RT** | 16.3±1.1 | 14.5±1.0 | 0.25 |
| **FR** | 12.1±0.8 | 11.2±1.0 | 0.44 |
| **LR** | 12.2±0.9 | 11.7±1.0 | 0.64 |
| **LOW score group (n=10)** | | | |
|  | **execution** | **imagery** | **p-value** |
| **RT** | 17.4±1.1 | 17.2±2.2 | 0.9 |
| **FR** | 14.1±0.8 | 15.3±1.4 | 0.35 |
| **LR** | 14.4±1.0 | 15.5±1.5 | 0.47 |
| Mean ± SEM are shown for rising on tiptoes (RT), forward reaching (FR) and lateral reaching (LR) execution and imagery tasks, respectively, followed by the corresponding paired *t* test p-value. | | | |

**Supplementary Table 2**

| **SUPPL. TABLE 2 \| Motor imagery vividness scores reported after each imagery tasks** | | | | |
| --- | --- | --- | --- | --- |
|  | **RTI** | **FRI** | **LRI** | **p-value** |
| HIGH | 4.0±0.2 | 3.9±0.2 | 3.0±0.2 | 0.9 |
| LOW | 2.2±0.2 | 2.5±0.3 | 3.0±0.3 | 0.08 |
| Imagery vividness was rated by using a 5-point scale adapted from Malouin et al. (2007). Mean ± SEM are shown for rising on tiptoes (RTI), forward reaching (FRI) and lateral reaching (LRI) imagery tasks, followed by the corresponding ANOVA p-value. | | | | |
